# Variation Revealed by SNP Genotyping and Morphology Provides Insight into the Origin of the Tomato

**DOI:** 10.1371/journal.pone.0048198

**Published:** 2012-10-31

**Authors:** Jose Blanca, Joaquín Cañizares, Laura Cordero, Laura Pascual, María José Diez, Fernando Nuez

**Affiliations:** Institute for the Conservation and Improvement of Agricultural Biodiversity, Polytechnic University of Valencia, Camino de Vera, Valencia, Spain; National Rice Research Center, United States of America

## Abstract

Tomato, *Solanum lycopersicum*, is divided into two widely distributed varieties: the cultivated *S. lycopersicum* var. *lycopersicum*, and the weedy *S. lycopersicum* var. *cerasiforme*. *Solanum pimpinellifolium* is the most closely related wild species of tomato.

The roles of *S. pimpinellifolium* and *S. l. cerasiforme* during the domestication of tomato are still under debate. Some authors consider *S. l. cerasiforme* to be the ancestor, whereas others think that *S. l. cerasiforme* is an admixture of *S. pimpinellifolium* and the cultivated *S. l. lycopersicum*. It is also not clear whether the domestication occurred in the Andean region or in Mesoamerica. We characterized 272 accessions (63 *S. pimpinellifolium*, 106 *S. l. cerasiforme*, 95 *S. l. lycopersicum* and 8 derived from hybridization processes) were morphologically and genetically using the SolCap platform (7,414 SNPs). The two species were distinguished in a PCA analysis and displayed a rich geographic structure. *Solanum lycopersicum* var. *cerasiforme* and *S. l. lycopersicum* were also differentiated in the PCA and Structure analyses, which supports maintaining them as different varieties. *Solanum pimpinellifolium* and the Andean *S. l. cerasiforme* were more diverse than the non-Andean *S. lycopersicum*. *Solanum lycopersicum* var. *cerasiforme* was morphologically and molecularly intermediate between *S. pimpinellifolium* and tomato. *Solanum lycopersicum* var. *cerasiforme*, with the exception of several Ecuadorian and Mexican accessions, is composed of the products of admixture processes according to the Structure analysis. The non-admixtured *S. l. cerasiforme* might be similar to the ancestral cultivars from which the cultivated tomato originated, and presents remarkable morphological diversity, including fruits of up to 6 cm in diameter. The data obtained would fit a model in which a pre-domestication took place in the Andean region, with the domestication being completed in Mesoamerica. Subsequently, the Spaniards took plants from Mesoamerica to Spain and from there they were exported to the rest of the world.

## Introduction

Tomato (*Solanum lycopersicum* L., formerly *Lycopersicon esculentum* Mill.), with a yield of 146 million tons in 2010, is the vegetable with the highest worldwide production (Faostat, http://faostat3.fao.org/home/index.html#VISUALIZE_TOP_20). Despite its economic importance, some aspects of its origin remain unclear. Most authors agree that *Solanum pimpinellifolium* L. is the closest wild species to the cultivated tomato, *S. lycopersicum* var. *lycopersicum* (*S. l. lycopersicum*), and that *S. lycopersicum* var. *cerasiforme* (*S. l cerasiforme*), a variety that usually grows in disturbed lands, is the ancestor of the cultivated variety. However, important aspects of the relationships between these species and varieties have yet to be completely clarified.

Tomato belongs to the Solanaceae family, *Solanum* L. genus, *Lycopersicon* section [1]. The wild relatives of the cultivated tomato are native to western South America, from northern Ecuador through Peru to northern Chile, including the Galápagos Islands. They are spread throughout diverse habitats that include the desert of the Pacific coast at sea level, the green inter-Andean valleys and mountainous Andean regions at an altitude of 3,300 meters. This peculiar ecological diversity in the Andean region has contributed to the variability of the tomato related wild species [2].

The phylogenetic relationships among the species included in the genus have been studied extensively using various molecular markers that include cpDNA [3], mtDNA [4], nuclear RFLPs [5] and AFLPs [6], [7]. Sequence data has also been employed: ITS rDNA [8], the GBSSI gene sequence by Peralta and Spooner [9] and two nuclear genes by Zuriaga et al. [7]. In these studies, *S. pimpinellifolium*, *S. l. cerasiforme* and the cultivated tomato consistently clustered together, showing their close genetic relationship.

The tomato, *S. l. lycopersicum*, is an almost strictly autogamous variety with a high degree of homozygosity. It is a perennial plant, although it is usually cultivated as an annual plant. Its stems are hairy and its leaves bipinnate. Its flowers usually have 5 petals, although it is also common to find flowers with 7 or more, and its styles are usually inserted. The tomato is cultivated because of its fleshy fruits. A wide range of varieties with fruits of different colors, shapes and sizes [10] are currently commercialized. It is commonly accepted that the genetic variability of the tomato is quite small due to different bottlenecks that took place during its domestication and diffusion [5], [11]–[14]. However, Rick [15], [16] reported variable degrees of diversity within tomato depending on the region of origin, with especially high levels in the Andean region.

The *S. l. cerasiforme* variety is mostly self-compatible and autogamous, although it shows variable rates of allogamy depending on the geographical region considered. It usually has red and rounded fruits that range from 1.5 to 3 cm in diameter. However, remarkable morphological variation has been found in different characters, including the fruit-related ones. Both flattened and ribbed fruits have been reported, with diameters ranging from 1.05 to 8 cm [17]. This variety grows spontaneously worldwide in tropical and subtropical regions [18]. It has been collected in a wide range of habitats that include deserts and very humid regions in altitudes that range from sea level to 2,400 m [2], although it prefers humid zones below 1,200 meters. It is widely distributed close to human-modified areas, such as irrigation canals, home gardens and orchards. It is sown in some rural areas, although it usually grows without human intervention, behaving as a weed. Due to its organoleptic quality, its fruits are frequently consumed fresh or used in sauces [17]. During our collecting expeditions, we also observed that it is used to feed poultry and other domestic animals.

The molecular variation of *S. l. cerasiforme* was studied by Rick and Fobes [16] using allozymes. They found a marked variability within the accessions from Peru and Ecuador when compared to accessions from Mesoamerica, North America, Europe and other South American regions. They also divided the Andean accessions into two groups: coastal and interior (east of the Continental Divide). In the accessions from the coastal region, all of the allozyme alleles were also present in the sympatric *S. pimpinellifolium*. Based on this, they argued that no bona fide *S. l. cerasiforme* exists in the coastal zone. Villand et al., using RAPDs [19], and Williams and St. Clair, using RFLPs and RAPDs [14], also found greater variability in tomato in the Andean region. More recently, Nesbitt and Tanksley [20] suggested that *S. l. cerasiforme* appears to be an admixture of *S. pimpinellifolium* and *S. lycopersicum* rather than a transitional step between wild and domesticated tomatoes, whereas Ranc et al. [21] proposed that this variety be divided into two groups, one being an admixture of *S. pimpinellifolium* and *S. lycopersicum* with the other being genetically close to the cultivated *S. l. lycopersicum*.

The wild species, *S. pimpinellifolium*, has a bushy growth type and inhabits the coastal regions of Ecuador, Peru and northern Chile. Its fruits are red and smaller than 1.5 cm in diameter. This species is mostly autogamous, although different degrees of allogamy have been reported in different geographical regions [22]. Although it is usually found below an altitude of 1,450 m [2], the members of this research team have collected it at 1,800 m above sea level in the Ecuadorian provinces of Loja and Azuay [23]. The natural range of *S. pimpinellifolium* encompasses such differing environments as the northern coastal Ecuadorian tropical rainforests and the Peruvian coastal desert [23]. In central and southern Peru, *S. pimpinellifolium* is restricted to cultivated fields, roadsides and dumping grounds, and its distribution is sparse, but in northern Peru and Ecuador it is found in wild and dense populations located in undisturbed areas [23], [24].

The genetic variation of *S. pimpinellifolium* has been studied with morphological characteristics [22], allozymes [16], [24], nuclear DNA gene sequences [25] and microsatellites [23], [26]. Studies that used morphological and allozymic variants showed different degrees of genetic variation and of autogamy rates, which ranged from 0 to 84% depending on geographic location [22]. The studies conducted with microsatellites revealed marked differences between the Peruvian and Ecuadorian accessions [23]. These analyses localized the region of maximum genetic diversity in Northern Peru.

*Solanum pimpinellifolium* and *S. lycopersicum* are interfertile, and a clear-cut classification is hampered by the existence of intermediate types [15], [17]. Fruit size has been used as the main criterion to classify *S. pimpinellifolium*, *S. l. cerasiforme* and *S. l. lycopersicum* [24]. However, it is now accepted that the demarcations between these clades are not straightforward and that other characters, such as leaf size and shape, flower and inflorescence sizes and the degree of pubescence should be taken into account [17].

A commonly accepted hypothesis for the domestication of the cultivated tomato is that *S. l. cerasiforme* originated in the Andean region, spread to Mexico as a weed, and became domesticated in Mexico, originating the first tomato cultigens that were later disseminated to the Old World [18], [27]. Rick and Holle [17], after evaluating all the morphological and molecular data available, also proposed a domestication in Mesoamerica, although they warned that no conclusive data existed and that an open mind would have to be maintained with regard to a possible domestication in the Andean region. There is also no unanimity regarding the involvement of *S. pimpinellifolium* and *S. l. cerasiforme* in the domestication process. Some authors have considered *S. l. cerasiforme* to be the ancestor of the tomato [18], [19], [28] as well as, alternatively, the result of an admixture of *S. pimpinellifolium* and *S. l. lycopersicum* [20].

Little historical evidence is available regarding the details of the domestication and subsequent diffusion processes. The first historical records date to the Spanish chroniclers, who were the first to document the tomato's cultivation and consumption in Mesoamerica [29]. Most authors maintain that the tomato arrived in Europe from Mexico. Isozymic variation studies indicate that European cultivars show a greater similarity to primitive Mexican cultivars and lines than to those of the Andean region [16]. Another piece of evidence of the Mesoamerican involvement in the European importation of the tomato is the name itself. The word *tomatl* existed in nahuatl, one of the native Mexican languages, and described plants bearing globose, juicy fruits. Terms derived from this word are still used in many languages to refer to the tomato. The Spanish people brought the tomato to Europe and, by the first half of the sixteenth century, clear evidence of this introduction appeared in European herbals. Later, and mainly from Europe, the crop diffused to the rest of the world through commercial routes and colonies. Before its return to the New World, the tomato had already gone through its first rounds of breeding [30].

In this study, a wide sample of 272 accessions covering the variation of *S. pimpinellifolium*, *S. l. lycopersicum* and *S. l. cerasiforme* were analyzed. A morphologically based classification was carried out as well as a SNP-based genotyping. The molecular analysis was based on the high-throughput genotyping platform prepared by the SolCap project [31], [32] and yielded a detailed representation of the molecular variation and structure of these species in addition to some insights about the origin of the cultivated tomato. The data collected also provided some clues as to whether *S. l. cerasiforme* is the tomato progenitor or is derived from a hybridization process between *S. pimpinellifolium* and tomato. Finally, two different birthplaces for the cultivated tomato may be earnestly considered: Mesoamerica and the Ecuadorian and Peruvian Amazonian region.

## Results

### Genetic structure

An analysis of the genetic variation present in 272 selected accessions (63 *S. pimpinellifolium*, 106 *S. l. cerasiforme*, 95 *S. l. lycopersicum* and 8 derived from hybridization processes) (Supporting Table S1) was carried out using the SolCAP tomato Infinium SNP array (Supporting Table S2). A total of 7,414 markers were used, of which 81.3% were found to be polymorphic among all accessions. The degree of polymorphism within *S. pimpinellifolium*, *S. l. cerasiforme* and tomato was 54.5%, 54.2% and 34.8%, respectively (Table 1).

**Table 1:** Polymorphism and heterozygosity indexes.

Species	Wide group	Limited group	Het. Exp.	Het. Obs.	% Het. Obs.	P(0.95)	Number of individuals
***S. l. cerasiforme***			**0.17**	**0.016**	**9.7**	**0.54**	**106**
	**Colombian**		**0.087**	**0.011**	**13.0**	**0.19**	**4**
	**Cusco**		**0.20**	**0.014**	**7.0**	**0.46**	**8**
	**Ecuadorian**		**0.18**	**0.028**	**15.2**	**0.48**	**24**
		Baeza	0.13	0.031	24.3	0.34	6
		Puyo	0.15	0.019	12.3	0.36	5
		Sucúa	0.14	0.060	41.6	0.36	6
		Zamora	0.074	0.002	2.8	0.16	5
	**Mesoamerican**		**0.095**	**0.015**	**15.9**	**0.27**	**32**
		Costa Rica	0.095	0.012	12.9	0.27	7
		Puebla	0.058	0.018	31.4	0.15	7
		Queretaro	0.027	0.003	12.4	0.061	5
		Salvador	0.072	0.013	18.5	0.18	6
		Sinaloa	0.028	0.009	31.0	0.070	4
		Yucatán	0.098	0.046	46.6	0.23	3
	**Northern Peruvian**		**0.15**	**0.014**	**9.5**	**0.49**	**16**
		Pasco	0.14	0.005	3.5	0.34	6
		San Martín	0.14	0.020	14.9	0.38	10
	**Other**		**0.091**	**0.009**	**9.4**	**0.21**	**22**
		Canary Islands	0.050	0.002	3.2	0.11	7
		World	0.096	0.012	12.1	0.32	15
***S. l. cerasiforme*** **×** ***S. l. lycopersicum***			**0.13**	**0.056**	**43.8**	**0.364**	**5**
***S. l. lycopersicum***			**0.11**	**0.013**	**11.9**	**0.35**	**95**
	**Mesoamerican**		**0.082**	**0.003**	**3.8**	**0.24**	**17**
		Cuba	0.027	0.004	13.9	0.040	2
		South American	0.065	0.002	2.8	0.12	3
		Oaxaca	0.063	0.001	1.6	0.14	5
		Yucatán	0.073	0.001	1.9	0.17	6
	**Modern Improved**		**0.14**	**0.041**	**29.4**	**0.39**	**25**
	**Non-Mesoamerican**		**0.072**	**0.002**	**2.6**	**0.20**	**50**
		Andalusia	0.060	0.009	14.5	0.16	6
		Bolivia	0.034	0.001	3.3	0.063	3
		Catalonia	0.056	0.001	2.1	0.14	6
		Canary Islands	0.062	0.001	1.5	0.13	4
		France	0.072	0.001	1.0	0.18	6
		Italy	0.066	0.001	2.0	0.16	6
		Old Improved	0.055	0.009	16.6	0.11	3
		Other	0.064	0.001	1.3	0.15	5
		Portugal	0.045	0.002	4.0	0.068	2
		USA	0.056	0.001	1.4	0.14	6
		Valencia	0.061	0.001	1.5	0.15	6
***S. lycopersicum*** **×** ***S. pimpinellifolium***			**0.32**	**0.107**	**33.3**	**0.65**	**3**
***S. pimpinellifolium***			**0.21**	**0.029**	**13.7**	**0.54**	**63**
	**Ecuadorian**		**0.071**	**0.013**	**18.6**	**0.23**	**16**
		Esmeraldas	0.044	0.011	24.3	0.092	5
		Manta	0.071	0.027	37.4	0.17	5
		Pedernales	0.044	0.004	9.3	0.12	6
	**Montane**		**0.13**	**0.011**	**8.6**	**0.34**	**12**
		Catamayo	0.096	0.017	17.3	0.19	3
		Jaen	0.091	0.014	15.7	0.21	4
		Machala	0.069	0.005	7.9	0.16	5
	**Peruvian**		**0.14**	**0.043**	**31.0**	**0.39**	**34**
		Coastal Piura	0.11	0.027	23.9	0.28	6
		Nazca	0.044	0.010	22.2	0.10	5
		Olmos	0.11	0.049	46.1	0.27	5
		Piura	0.12	0.069	57.3	0.32	6
		Sullana	0.13	0.067	51.2	0.35	6
		Trujillo	0.076	0.036	47.9	0.20	6

Wide group: Broad geographic group.

Limited group: Narrow geographic group.

Expected heterozygosity assuming Hardy-Weinberg equilibrium and corrected for sampling bias.

Observed heterozygosity.

Het. Obs./Het. Exp. * 100.

Polymorphism. Percentage of markers with a frequency of the most common allele bellow 0.95.

**Figure 1:**
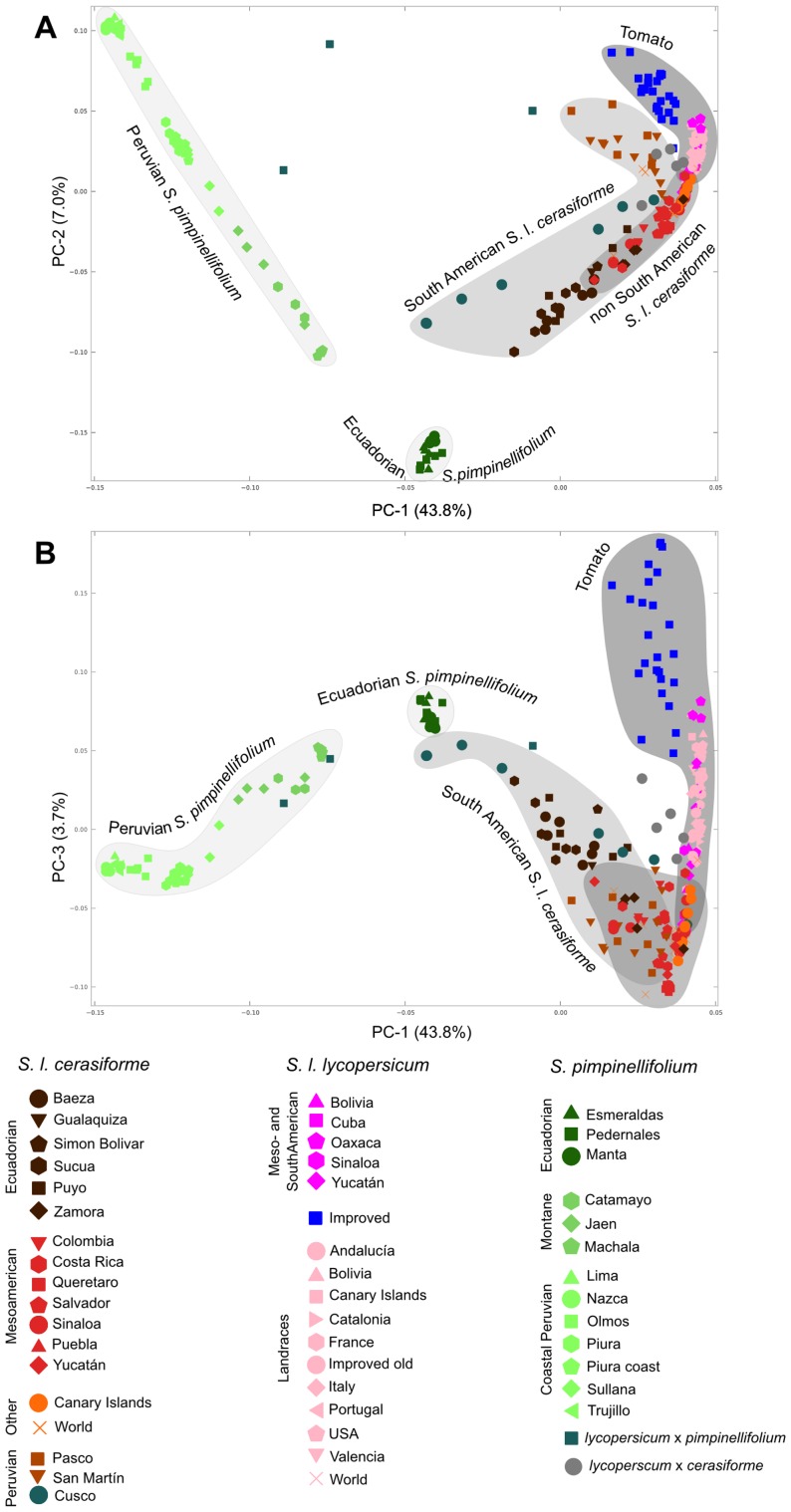
PCA analysis of all samples. In panel A the projection along the first and second principal components of the PCA analysis carried out with the SNP genotypes is represented. Panel B corresponds to the same PCA analysis, but in this case the samples are projected along the first and third principal components. Every axis label includes the percentage of the eigenvalues corresponding to that principal component. The colors and marker shapes represent the different, mainly geographical, groups in which every species and variety has been divided, and which are detailed in the legend. This division matches the one stated in the column “Limited Group” of Supporting Table S1.

A principal component analysis (PCA) was carried out with the smart-PCA [33] software in order to study the genetic relatedness of the accessions (Figure 1, panels A and B). Three non-overlapping groups were clearly observed in this PCA. The bulk of the Peruvian *S. pimpinellifolium* accessions comprised one of the groups. Another cluster included the northern Ecuadorian *S. pimpinellifolium* samples and the third was composed of *S. lycopersicum*. Also worthy of mention is the fact that the intermediate location between the *S. pimpinellifolium* and *S. lycopersicum* clusters was occupied by the three southern Peruvian accessions morphologically classified as hybrids between the two species. In parallel, an AMOVA with three clusters (*S. pimpinellifolium*, *S. l. cerasiforme* and *S. l. lycopersicum*) and their respective geographical subgroups was carried out using the Arlequin software [34]. Differences among the taxa accounted for 48.6% of the variation, whereas 18.4% was due to geographical subgroups within those taxa and 33% corresponded to the geographical subgroups irrespective of their taxa.

The main *S. pimpinellifolium* group showed a clear substructure in which the coastal and montane accessions were clearly segregated. From here on we will refer to these two clusters as Peruvian and Montane *S. pimpinellifolium*, respectively. The groups included in the Montane region were Machala, Catamayo and Jaen and were all located in the western and central Andean valleys. These groups showed a clear latitudinal cline that separated the accessions along a geographical north-to-south axis that was clearly seen in the PCA representation.

In the *S. lycopersicum* PCA group, the *S. l. cerasiforme* and cultivated tomato subgroups were almost completely segregated. The bulk of the Andean *S. l. cerasiforme* was distributed in a latitudinal cline that comprised the Ecuadorian and northern Peruvian accessions. The *S. l. cerasiforme* accessions in the Cusco region (southern Peru), which was somewhat closer to *S. pimpinellifolium*, represent the only exception to this cline. By contrast, the non-Andean *S. l. cerasiforme* occupied an intermediate position between the San Martín and Zamora *S. l. cerasiforme* groups and the traditional tomato varieties and heirlooms. The relationship between these *S. l. cerasiforme* clusters was best appreciated when all three of the first PCA components were taken into account. Whereas in the representation of the first and second components the tomato and northern Peruvian *S. l. cerasiforme* distributions were close and parallel (Figure 1, panel A), in the projection of the first and third components both clusters lay quite apart (Figure 1, panel B).

**Figure 2:**
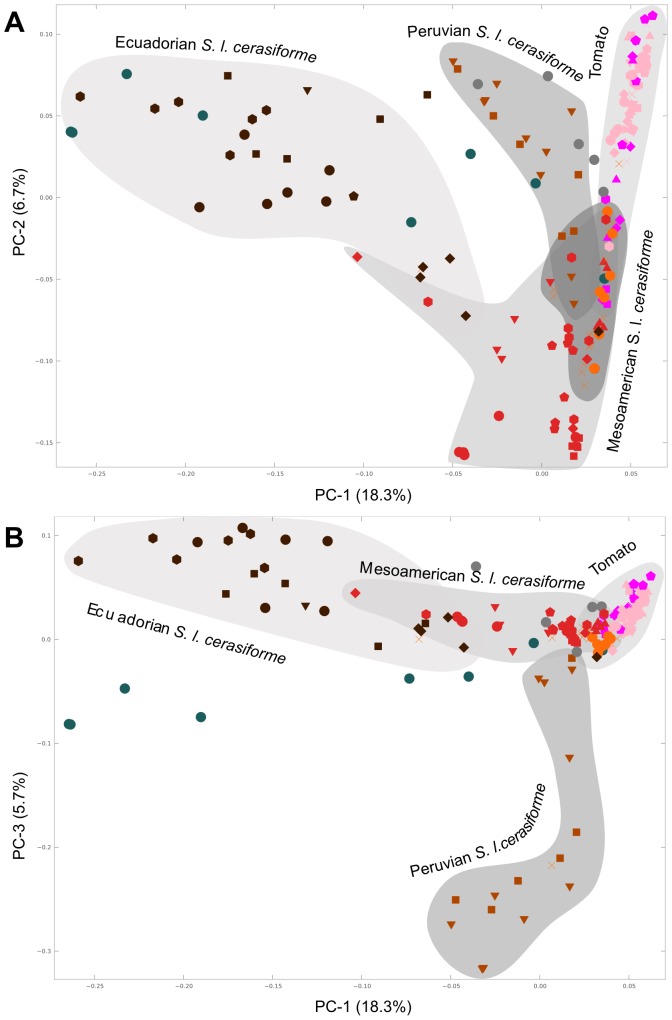
PCA analysis of *S. lycopersicum*. PCA analysis of the *S. lycopersicum* samples. In this case, as in Figure 1, panels A and B represent projections along different principal components. The colors and marker shapes represent the different, mainly geographical, groups in which *S. lycopersicum* has been divided and which are specified in the legend of Figure 1.

For a more detailed view of the relationship between *S. l. cerasiforme* and tomato, a new PCA was carried out without *S. pimpinellifolium* (Figure 2, panels A and B). In this new analysis, the northern Peruvian and Ecuadorian *S. l. cerasiforme* formed two separate clusters, with the Mesoamerican *S. l. cerasiforme* being located between them. This latter cluster partially overlapped with the one formed by the traditional tomato varieties, and it is in this overlap that the non-American *S. l. cerasiforme* was located. All of these clear clusters contrasted with the lack of grouping of the southern Peruvian *S. l. cerasiforme*, which was found to be scattered all over the PCA. Finally, the accessions that were morphologically classified as intermediate between *S. l. cerasiforme* and tomato were found halfway between the corresponding genetic groups. The AMOVA analysis was also repeated, removing *S. pimpinellifolium*. In this case, the variation that corresponded to differences between the *S. l. cerasiforme* and *S. l. lycopersicum* varieties was 11.5%, where 22.0% was due to differences among different geographical groups within the varieties and 66.5% to geographical groups irrespective of their varieties.

A third PCA that included only traditional tomato varieties was carried out. In it the Mesoamerican tomatoes were clearly separated from the rest, but among the non-American tomatoes only a mild geographic structuring was found (data not shown).

**Figure 3:**
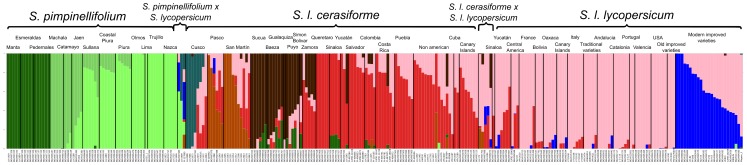
Ancestries inferred by the Structure analysis. Representation of the ancestries inferred for each sample by a Structure analysis carried out with 9 ancestral populations. Each bar corresponds to one accession and the color composition matches the ancestral population ancestry determined by Structure for that sample. The accessions belonging to each geographical group are separated by black bars and the captions specify the different geographical groups as they were assigned in the passport data included in Supporting Table S1.

**Figure 4:**
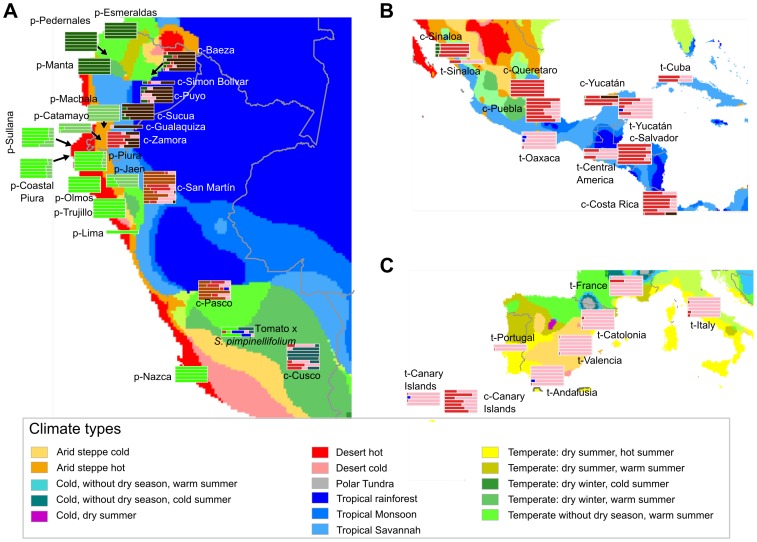
Geographical distribution of the Structure ancestries. The ancestries calculated by the Structure analysis are clustered by geographical group and represented at the corresponding geographical location. The ancestries' bar color matches those shown in Figure 3. The different colors of the geographical background correspond to the Köppen-Geiger climatic classification. The different climate types are detailed in the legend.

In addition to the PCA, a Bayesian-based population assignment allowing admixture was carried out using the Structure software [35]. After representing the likelihood for different numbers of populations, ranging from 2 to 19, a partition using 9 populations was chosen to be further analyzed (Supporting Figure S1). The ancestries clearly differentiated *S. pimpinellifolium*, *S. l. cerasiforme* and the cultivated tomato (Figure 3). Within these species and varieties, a noticeable substructure was found. For instance, whereas the traditional tomato varieties' ancestries corresponded mostly to just one Structure population, the modern, improved materials showed an admixture of the traditional varieties along with a modern component. In *S. l. cerasiforme* and *S. pimpinellifolium*, the bulk of the substructure found by the Bayesian analysis was linked to geography. To study this relationship, a representation of the ancestries on a geographic map was prepared (Figure 4). The geographic structure found in this analysis is in agreement with the previously described PCA analysis. For instance, *S. pimpinellifolium* is divided into three groups: Peruvian, Montane and northern Ecuadorian, although it should be noted that between the Peruvian and Montane groups a continuous variation was found with some northern Peruvian populations, such as Sullana and Coastal Piura.

In *S. l. cerasiforme*, a prominent geographic division between the following regions was also found: Ecuador, northern Peru, southern Peru, Mesoamerica and non-American. In *S. l. cerasiforme*, the signs of admixture abound. The bulk of Andean *S. l. cerasiforme* was comprised of plants that showed clear signs of admixture. The composition of the populations proposed by the Structure software was different within each geographic region, but admixture was found in almost all of them. The only geographical group of Andean *S. l. cerasiforme* with little admixture was found in the Sucúa region. In this region, the plants showed no admixture with *S. pimpinellifolium*, or if they did, it was a very small amount. Similarly, some of the *S. l. cerasiforme* accessions from San Martín also showed no admixture.

The Structure analysis divided Mesoamerican *S. lycopersicum* into two populations: one mostly made up of *S. l. cerasiforme* and the other of traditional tomato varieties, although, again, some intermediate groups were found showing admixture, such as the *S. l. cerasiforme* from Costa Rica and Puebla or the tomato from Cuba and Yucatan. The non-American *S. l. cerasiforme*, like those from the Canary Islands, also showed admixture between these two Structure populations, whereas the traditional tomato varieties from Europe, the USA and Asia were quite homogeneous and only showed one Structure population corresponding to the traditional Mesoamerican varieties.

### Climate

Strikingly different ecological and climatic regions are found in the Andean region inhabited by *S. pimpinellifolium* and *S. l. cerasiforme*, ranging from the Peruvian coastal desert to the eastern Ecuadorian tropical rainforest. It has been noted by previous studies that the genetic structure of *S. pimpinellifolium* correlates with these climatic differences [23], so a Köppen-Geiger climatic classification [36] was represented along with the ancestries calculated by the Structure software in a geographic map (Figure 4).

In *S. pimpinellifolium*, the relationship between climate and geographic genetic differentiation was clearly noticeable. The three groups found by molecular analyses corresponded quite clearly to three different climatic regions: a desert on the Peruvian coast, another arid hot steppe in the montane region and finally a temperate region with no dry season in northern Ecuador. The accessions collected in this latter region, whose climate is the most different from the dry climate typical of the region of maximum diversity of *S. pimpinellifolium*, were genetically and morphologically also the most divergent.

A similar division was found in the eastern Andean *S. l. cerasiforme*. The northern Ecuadorian populations thrive in a rainforest climate while their Peruvian counterparts occupy either a region of tropical savannah or one with a temperate climate with less rainfall and no dry season. In Mesoamerica, a climatic, latitudinal cline of humidity was found to run from the south, where the climate is similar to that of eastern Peru, up to the temperate and arid regions of northern Mexico. Finally, the temperate Mexican climate resembles the temperate Mediterranean climate, with dry summers like those of Spain and Italy.

### Polymorphism and heterozygosity

Several indexes related to diversity and heterozygosity were calculated for the different species, varieties and geographical groups (Table 1). The polymorphism in *S. pimpinellifolium* was similar to that of *S. l. cerasiforme* (0.54) and higher than that of the cultivated tomato (0.35). The polymorphism found in the different groups within these taxa also showed marked differences. For instance, in *S. pimpinellifolium*, the geographical groups close to the Piura region showed the highest polymorphism (Piura 0.32 and Sullana 0.34), whereas the variability in this species was reduced in the northern (e.g. Esmeraldas 0.09) and southern latitudes (e.g. Nazca 0.10).

Prominent differences in variability were also found within *S. l. cerasiforme*. While the polymorphism found in *S. l. cerasiforme* from different Andean regions (0.50, 0.43 and 0.58) was similar to that found in *S. pimpinellifolium*, the genetic polymorphism in Mesoamerica was much lower (0.26). A similar degree of polymorphism (0.24) was found in the traditional cultivated varieties from the same region as well as in the heirlooms from the rest of the world (0.20). In contrast to this low polymorphism a higher level of polymorphism was detected in the modern, improved materials (0.39).

The ratio of observed to expected heterozygosity (expressed as a percentage) was only slightly higher in *S. pimpinellifolium* (13.7%) than in *S. l. cerasiforme* (9.7%) and *S. l. lycopersicum* (11.9%), but marked differences between regions were found for this parameter. *In S. pimpinellifolium*, the maximum heterozygosity was associated with the regions with the highest polymorphism, Piura and Sullana (57.3%), whereas heterozygosity was much lower in the north (e.g. Machala (7.9%) and Pedernales (9.3%)) and in the south (Nazca (13.7%)).

In *S. l. cerasiforme*, regional variation in heterozygosity was also found with values as high as 40% in Sucúa and Yucatán and as low as 3% in Pasco and the Canary Islands. In the cultivated tomato, all regions had low heterozygosity, ranging from 1 to 4% for all traditional varieties, while the modern, improved varieties, which included some commercial hybrids, had higher heterozygosity (16.6%).

### Morphology

**Figure 5:**
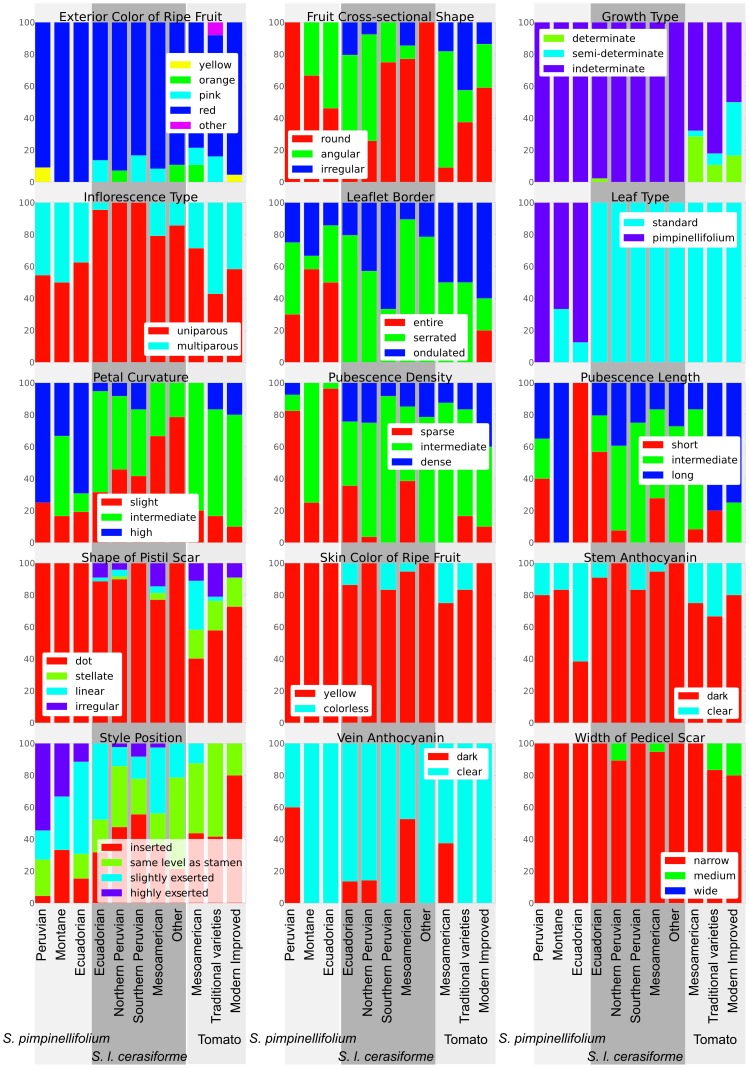
Qualitative morphological characters. The distribution of the different qualitative morphological characters throughout the groups in which the different species and varieties have been divided is represented. Each chart corresponds to a different character and each bar to a different group. The percentages are calculated over the number of plants found to have every type of the character. The accession grouping is mainly geographical and is listed in the “Wide Group” column of Supporting Table S1.

A representation of the qualitative morphological data was prepared for the different taxa and geographical regions (Figure 5). For the majority of characters, all observed types were present in *S. pimpinellifolium*, although important differences in frequency were observed when compared to *S. lycopersicum*. For instance, both the standard ‘tomato’ and ‘pimpinellifolium’ types of leaf (as depicted in the IPGRI tomato descriptors [37]) were found in Ecuadorian *S. pimpinellifolium*, although only the ‘pimpinellifolium’ type was present in the Peruvian region of maximum variability of the species. By contrast, the ‘tomato’ type predominated in *S. lycopersicum*. In the case of the style position, all types were found in every region occupied by *S. pimpinellifolium*, ranging from highly exserted to inserted. However, in *S. l. cerasiforme*, the style tended to be somewhat more inserted, whereas in the cultivated tomato the more inserted types were more common.

In contrast, some characters showed types that were only present in *S. lycopersicum* and were completely missing from *S. pimpinellifolium*. In most of these characters, the type exclusive to the cultivated tomato was also present in *S. l. cerasiforme*, as is the case of the irregular cross-sectional fruit shape, the irregular shape of the pistil scar, the colorless skin color of the ripe fruit and the medium width of the pedicel scar. There were few morphological characteristics exclusive to the cultivated variety. Only for growth type did semi-determinate and determinate types not appear in either *S. l. cerasiforme* or *S. pimpinellifolium*.

**Figure 6:**
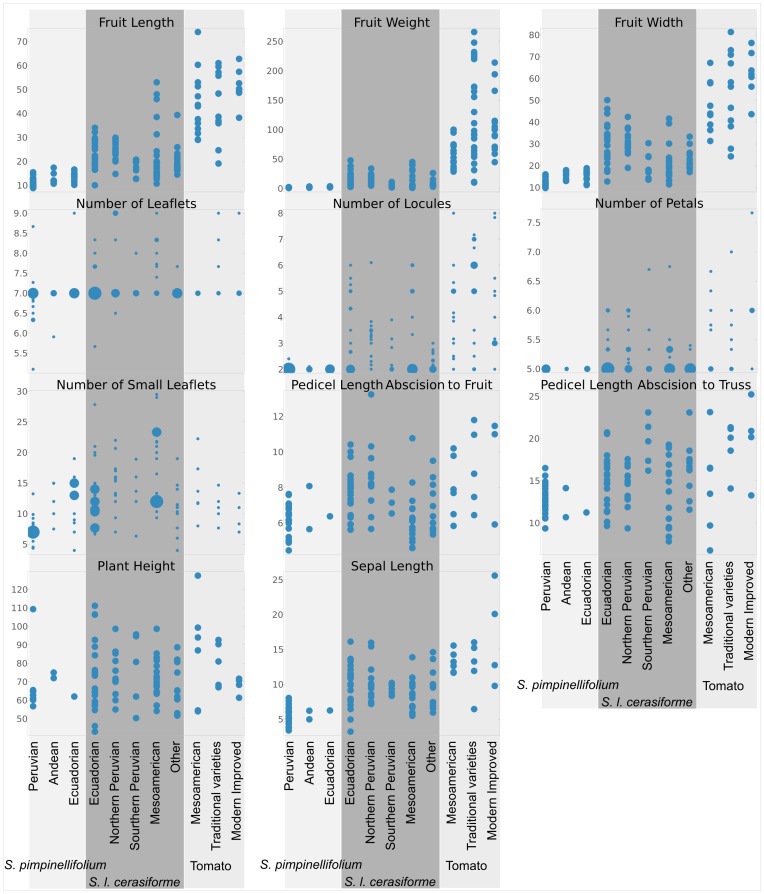
Quantitative morphological characters. The distribution of values for different quantitative morphological characters is represented for the groups in which the different species and varieties have been divided. Each chart corresponds to a different character and each column to a different group. In the continuous characters, each point in the scatter plots represents the mean value of the character for that accession, whereas in the discontinuous ones, the number of accessions that have the same value for the given character is represented by the diameter of the mark in the scatter plot. The accession grouping is the same as that used in Figure 5, is mainly geographical and is listed in the “Wide Group” column of Supporting Table S1.

Another graphical representation was prepared for the quantitative morphological data (Figure 6). In this case, several characters showed little or no differences between the taxa, such as plant height or pedicel length measured from abscission to fruit. However, other characters, for instance those related to fruit size (fruit length, fruit weight, fruit width and number of locules), were notably different between *S. pimpinellifolium*, *S. l. cerasiforme* and tomato. *Solanum lycopersicum* var. *cerasiforme* showed intermediate values between the wild species and the cultivated variety for all of these fruit characters. For instance, while only two locules were usually found in *S. pimpinellifolium*, 6 locules were common in almost all of the *S. l. cerasiforme* groups, and the number of locules in tomato was frequently higher. A similar pattern was found for sepal length, which was much higher in *S. lycopersicum* than in *S. pimpinellifolium*. In this latter case, the traditional tomato varieties had lengths similar to those of *S. l. cerasiforme*. The modern, improved materials had the longest sepals.

A canonical discriminant analysis (CDA) was carried out with the quantitative and ordinal characters to assess which characters were the most discriminant between the three taxa (Supporting Figure S2). The first two canonical variables obtained accounted for 78.5% of the variation. The first canonical variable (58.4%) was mainly composed of the following characters: fruit length (26.0%), petal curvature (13.2%), stem pubescence density (9.6%) and number of petals (9.5%).

A Mantel test [38] gave the correlation between the morphological and genetic distances. The Euclidean distance was used for the quantitative and ordinal morphological characters, the Jaccard distance for the binary and nominal morphological characters and the Nei minimum distance for the genetic data. A correlation of 0.36 was obtained with a p-value of 0.001.

## Discussion

### Classification of the Accessions

In this study, 272 accessions were analyzed. It is of particular interest to note that some of the accessions collected by the COMAV Institute represent regions that are traditionally neglected, such as the *S. pimpinellifolium* from the northern Ecuadorian coast or the *S. l. cerasiforme* from the eastern Andean Ecuadorian lowlands. These materials populate regions with ecologies that are complementary to those usually considered for these species.

In parallel to the molecular analysis, a morphological classification based on the criteria proposed by Rick and Holle [17], and similar to the one proposed by Luckwill [39], was carried out. In addition to fruit size, the criteria included other characters, such as leaf characteristics, inflorescences and flowers, as well as the degree of anthropomorphic impact on the habitat. This set of morphological criteria was found to generate groups that were genetically more coherent, according to the PCA and Structure analyses, than those obtained using the more straightforward classification system based mainly on fruit size. The CDA analysis gave importance to fruit length, but it also took into consideration other characters to distinguish between the species. This concordance between the molecular and morphological classifications was also observed by Rick and Holle when they proposed this classification system [17]. The reexamination of the given classifications for the different accessions is particularly important within *S. lycopersicum*, as different authors have used different criteria to distinguish the two varieties that comprise this species, with some authors even modifying their criteria from study to study. For instance, in 1958 Rick [15] classified accessions as *S. l. lycopersicum* that, with the newer rules, proposed by himself in 1990 [17], would have been classified as *S. l. cerasiforme*. The application of these more nuanced criteria lead to the reclassification of several accessions from Peru and Mexico as *S. l. cerasiforme* when, according to their fruit sizes, they would otherwise be considered to be *S. l. lycopersicum*. Maintaining the division of *S. lycopersicum* in the two *S. l. cerasiforme* and *S. l. lycopersicum* varieties was recently contested by Peralta and Spooner [40], who proposed to eliminate it. We consider that, due to the molecular (PCA) and morphological (CDA) differentiation observed in this study, this distinction is still useful.

The correlation between the genetic and morphological distances demonstrated by the Mantel test (0.36) could be considered moderate. This result is to be expected because, while there is a marked differentiation between the three main groups, within those groups the correlation between the morphological and genetic distance should not be too high. For instance, within *S. l. lycopersicum*, the different cultivars show considerable morphological differentiation even though their genetic differentiation is low [5]. This observation was made, for instance, in a collection of traditional Italian tomato varieties [41].

Another potential classification pitfall occurs when distinguishing between traditional varieties and modern improved materials. It is not possible to do this classification based only on the passport data recorded at the collection site. For instance, some accessions collected in traditional markets or in small gardens could include materials derived from modern improved cultivars. Differentiating between traditional and modern varieties can be improved by employing a morphological classification that takes into account characters such as fruit size uniformity, set sequence uniformity, core size and the presence of scars on the fruit. In fact, in this study, once the morphological characterization was completed, several accessions that had been previously considered to be traditional varieties due to their passport data were classified as modern improved materials. These reclassified accessions included, among others, all of the traditional tomato varieties from Ecuador. The invasion of the traditional markets by improved foreign materials was already recorded as early as the 1950s [15]. Regarding this classification, it is worthy of note that even when the characters mentioned were considered, certain accessions that were classified as traditional varieties showed, according to the Structure analysis (Figure 3), a small amount of the modern genome. Both the reclassification of *S. l. lycopersicum* to *S. l. cerasiforme* as well as the transition from traditional cultivars to modern materials have deprived the Andean region of traditional tomato varieties, as all of the corresponding accessions have ended up as modern or as *S. l. cerasiforme*.

### Genetic and morphological variability

Genetic variability was not uniform among the different populations of the different taxa. In *S. pimpinellifolium*, the geographic structure of the genetic variability was quite evident, and was tightly correlated with the climate characteristics of each region: the Peruvian coastal desert, the humid and temperate northern Ecuadorian forests and the montane regions of the Andes. These geographically related differences on the level of diversity already described when *S. pimpinellifolium* was studied with allozymes [24] and microsatellites [23], and is indicative of the importance of the climatic and ecological compartmentalization of the habitats occupied by the wild species.

In *S. l. lycopersicum*, the polymorphism was lower than in the other groups, especially in the non-American cultivars. This result is compatible with previous studies, for instance a recent study by Mazzucato et al. that also found low diversity in the traditional old-world tomato varieties [41].

In *S. l. cerasiforme*, the polymorphism found in different geographical regions was also very different: 0.48 in Ecuador, 0.49 in northern Peru, 0.27 in Mesoamerica and 0.20 in the non-American samples. Rick and Fobes [16] also evaluated the polymorphism in this variety and found results consistent with those presented in the current study: the Andean samples were moderately polymorphic, whereas the extra-Andean ones were remarkably less variable. The studies done with microsatellites found an intermediate diversity for *S. l. cerasiforme* between *S. pimpinellifolium* and *S. l. lycopersicum* [21], a result that is also compatible with that obtained in the current study.

The morphological diversity of *S. l. cerasiforme* was also high. The Andean *S. l. cerasiforme* had a morphology that was very different from the typical small, uniform fruits of *S. l. cerasiforme*. In this region, a high morphological variation was found which encompasses that found in *S. pimpinellifolium* as well as a significant part of the variation typical of the traditional tomato varieties. Especially striking is the variation found in fruit shape and size, which includes very small fruits, almost indistinguishable from those of *S. pimpinellifolium*, and large fruits that could easily be classified as small tomatoes. In fact, as has already been noted, in 1958 Rick [15] classified accessions as traditional Andean tomato varieties that are, according to the morphological descriptions and figures presented in that article, indistinguishable from the *S. l. cerasiforme* presented in this study.

### Structure of *S. l. cerasiforme* diversity

Even though *S. l. cerasiforme* appears as a coherent group in the PCA when compared to *S. pimpinellifolium* and *S. l. lycopersicum*, it also shows a marked substructure, especially in the Andean region. In the PCA carried out without *S. pimpinellifolium*, three different Andean groups could be defined in *S. l. cerasiforme*: Ecuador, northern Peru and southern Peru. Moreover, a marked geographical substructure is evident even within these broad regions. For instance, a clear latitudinal cline is observed in Ecuador, both in the PCA and in the Structure analyses (Figures 1 and 4). In some of these regions, *S. l. cerasiforme* appears to be derived from hybridization processes between different populations of *S. l. cerasiforme*, *S. pimpinellifolium* and *S. l. lycopersicum*. For example, in Zamora, *S. l. cerasiforme* seems to be, according to the Structure results, an admixture between northern *S. l. cerasiforme* populations and Mesoamerican *S. lycopersicum*. However, the possibility of this population being the result of the admixture of two Andean populations, one of which would be related to the origin of the Mesoamerican *S. l. cerasiforme*, cannot be ruled out. Other south Peruvian *S. l. cerasiforme* accessions are, according to the molecular data, the result of a hybridization between *S. pimpinellifolium* and *S. lycopersicum*. Some of these accessions were even morphologically classified as having a hybrid origin. This extended hybridization process could be responsible in part for the high genetic variability detected in the Andean *S. l. cerasiforme*, equivalent to that found in wild *S. pimpinellifolium*. These admixtures have been previously detected in several molecular-based studies carried out with isozymes [17], DNA sequences [20] and microsatellites [21]. Nesbitt and Tanksley [20] proposed that *S. l. cerasiforme* is in fact the product of the hybridization between wild *S. pimpinellifolium* and *S. l. lycopersicum*, while Ranc et al. [21] divided it into two groups, one being an admixture and the other being very close to *S. l. lycopersicum*.

The frequent admixtures between the weedy *S. l. cerasiforme* and wild *S. pimpinellifolium* found in the eastern lowlands of the Andes, especially in southern Peru, deserve some explanation as these eastern inland regions are not the usual habitat of *S. pimpinellifolium*, which is most commonly found west of the Andes. Three possible mechanisms could be at play to create such hybridizations. First, it is worthy of note that although *S. pimpinellifolium* is not habitually found in the high Andes, there are natural corridors, composed of a network of valleys, that cross the Andes, and there are populations of *S. pimpinellifolium* that inhabit some of those valleys. One such corridor exists in the Jaen region, and some of the *S. pimpinellifolium* accessions analyzed in this study are located there, deep inside the Andes, close to the San Martín *S. l. cerasiforme* populations. The other possible mechanisms that would facilitate these hybridizations between western and eastern plants could be related to human activity. In some of the collecting expeditions carried out on the eastern Andean slopes, Nuez observed *S. pimpinellifolium* living as a weed. For instance, he found a weedy *S. pimpinellifolium* in a cultivated field of *Piper nigrum* in the Morona-Santiago region in eastern Ecuador. The farmer reported having migrated along with his black pepper seeds from the coastal area. These plants may have participated in hybridization events with the native *S. l. cerasiforme*. Finally, another mechanism that might explain this hybridization involves the participation of the cultivated tomato. Hybrids between the cultivated tomato and *S. pimpinellifolium* may have occurred in the coastal regions, and from there these hybrids would have been transported to domestic gardens located in the eastern regions ready to be hybridized with the native *S. l. cerasiforme* [15].

Despite the abundance of admixture in *S. l. cerasiforme*, not all accessions belonging to this variety appear to be the result of an extensive hybridization process. For instance, the accessions from the Sucúa region in Ecuador and some from San Martín in northern Peru do not seem to be admixtures according to the Structure analysis, although in several a small contribution of the northern Ecuadorian *S. pimpinellifolium* genome could be detected. If we ignore the clear southern Peruvian admixtures, the Sucúa accessions are also the closest *S. l. cerasiforme* accessions to *S. pimpinellifolium* in the PCA. Both their lack of admixture and their closeness to the wild species might indicate that the *S. l. cerasiforme* accessions of the Sucúa region might be similar to the *S. l. cerasiforme* that inhabited the region before foreign tomatoes arrived in the Andean region. It is remarkable that the *S. l. cerasiforme* from this region also has high polymorphism and high heterozygosity that are equivalent to those found for *S. pimpinellifolium* in Piura, its region of maximum variability and allogamy [23], [24].

Finally, the extensive admixture and marked geographical structure found in *S. l. cerasiforme* might seem at first sight incompatible, but it is reasonable to hypothesize that the admixture process has been going on for a long time in the Andean region, but at a pace slow enough to maintain the observed geographical differentiation. Rick and Holle also found a marked geographical structure in the Andean *S. l. cerasiforme* and proposed that it had developed over centuries if not millennia [17].

### *S. l. lycopersicum*: origin and diffusion

There are still two alternative hypotheses about the relationship between *S. pimpinellifolium* and *S. l. cerasiforme*: Jenkins [27] and Rick [16] proposed that *S. l. cerasiforme* originated from *S. pimpinellifolium* and, alternatively, Nesbitt and Tanksley proposed a hybrid origin of *S. l. cerasiforme* from *S. pimpinellifolium* and tomato [20].

The data obtained in the present study agree with *S. pimpinellifolium* as the origin of *S. l. cerasiforme*. In the PCA, the northern Ecuadorian accessions of *S. l. cerasiforme*, including the non-admixtured ones, were close to the wild northern Ecuadorian *S. pimpinellifolium*. Despite the PCA result, due to the lower diversity and heterozygosity of the northern Ecuadorian *S. pimpinellifolium*, we would not hypothesize that the coastal Ecuadorian *S. pimpinellifolium* was the only origin of the highly diverse eastern *S. l. cerasiforme*. The highly diverse Ecuadorian *S. l. cerasiforme* may have originated with the participation of the Montane *S. pimpinellifolium* from Machala, Ecuador, with the possible participation of other regions. Rick and Holle [17] also proposed that the eastern Andean *S. l. cerasiforme* might have originated from the coastal *S. pimpinellifolium*. This process could have been favored by the high rainfall typical of both slopes of the Ecuadorian Andes. The wild plants, adapted to high humidity environments on the western Andean slopes, could have easily migrated to the wet eastern slopes once the landscape was modified by man. This high rainfall characteristic of eastern Ecuador could also have prevented the invasion of foreign cultivated tomatoes and could have favored the isolation of some ancestral Ecuadorian *S. l. cerasiforme* populations. Rick [42] and Nuez (personal observation) reported that it is not possible to cultivate modern tomato varieties in these regions due to their susceptibility to fungal attacks. In fact, the tomatoes cultivated by locals are local *S. l. cerasiforme*. It is worthy of note that Ecuadorian *S. l. cerasiforme* is a valuable breeding resource that has been neglected despite its high variability and the unusual habitat that it occupies.

Once *S. l. cerasiforme* had emerged in eastern Ecuador, it may have migrated to Mesoamerica as a weed or by direct human action. The closest Andean *S. l. cerasiforme* to that of Mesoamerica was, according to the PCA, the one located in the Zamora, San Martín and Pasco regions. We cannot assume, however, that this resemblance is necessarily due to an ancestral relationship because the Structure analysis shows that most of the accessions from these regions originated from recent admixture processes. It is clear, though, that this migration implied a drastic reduction in polymorphism and in heterozygosity, as can be observed in Table 1. This lower diversity of Mesoamerican *S. l. cerasiforme* has already been described in previous studies [16]. This pattern of diversity is in agreement with the general model of colonization proposed by Vavilov (reviewed by Jenkins [27]), which involves both a bottleneck that reduces the diversity and a selection of the more autogamous individuals, favoring spreading in regions with a scant presence of congeners.

Nowadays, *S. l. cerasiforme* inhabits wide regions of the tropics and subtropics outside of its pre-Columbian range [18]. The morphological and genetic variation of these non-American plants is, according to the present study, quite narrow. This is also supported by a previous study carried out using RAPDs [19]. Two hypotheses have been proposed to explain the origin of this worldwide invasion: they could have derived from American *S. l. cerasiforme* or, alternatively, they could have originated from traditional tomato varieties grown worldwide. In the latter case, the cultivated tomatoes abandoned to a feral way of life would have reverted in morphology to an original pre-domesticated state. In the PCA and Structure analyses, little difference, besides a lower diversity found outside America, can be found between the Mesoamerican *S. l. cerasiforme* and that which inhabits the subtropical regions outside America. This similarity fits better with the first hypothesis, the American *S. l. cerasiforme* spread throughout the tropics, although we would assume that some genetic flow between the cultivated tomatoes and *S. l. cerasiforme* is probably still going on in the regions in which both varieties coexist. Jenkins in 1948 [27] already proposed the difficulty that cultivated tomatoes would have in reverting back to a phenotype similar to that of *S. l. cerasiforme*, and that the spread of this variety, which is usually associated with human-disturbed areas, likely originated from within its pre-Columbian range.

### *S. l. cerasiforme*: origin and diffusion

As has already been stated, *S. l. cerasiforme* is commonly considered the ancestor of the cultivated tomato [18], although two other alternative hypotheses have been proposed: one suggested by Brücher in 1969 [43], [44] and another by Nesbitt and Tanksley in 2002 [20]. Brücher proposed *Lycopersicon humboldtii* (Willd.) Dunal as the tomato ancestor. According to Brücher, *L. humboldtii* would have been a wild species, different from *S. l. cerasiforme*, with fruits between 4 and 6 cm, and with leaves similar in shape to those of *S. pimpinellifolium*. This species was originally collected by Humboldt and later by Brücher in the Department of Aragua, Venezuela, a region of high rainfall. The classification of the Brücher collections as *L. humboldtii* was questioned by Teppner [45]. According to Teppner, this species was originally described by Willdenow as small-fruited, with fruits with diameters of around 1 cm, which would correspond in a modern classification to *S. l. cerasiforme*. We consider that both the large- and small-fruited plants could both in fact be classified as *S. l. cerasiforme*. We have observed accessions of this variety with both large and small fruits and with different leaf types despite being genetically homogeneous and belonging to the same population (Figures 5 and 6). The morphological range of variation of *S. l. cerasiforme* encompasses plants similar to those described by Brücher and to the typically small-fruited *S. l. cerasiforme* found in non-American subtropical regions. This would resolve the controversy proposed by Brücher as to the species from which the cultivated tomato originated.

Alternatively, Nesbitt and Tanksley proposed that *S. l. cerasiforme* appeared to be an admixture of wild and cultivated tomatoes rather than a transitional step from wild to domesticated tomatoes [20]. However, the molecular and morphological data collected in this study also agree with *S. l. cerasiforme* as the ancestor of the cultivated tomato. The following evidence supports both hypotheses: A) The PCA clearly showed that *S. l. cerasiforme* occupies an intermediate position between *S. pimpinellifolium* and *S. l. lycopersicum*, although it is closer to the latter. B) *S. l. cerasiforme* showed intermediate morphological characteristics in the characters that differentiate *S. pimpinellifolium* and *S. l. lycopersicum*. Especially notable in this regard are the characters related to fruit size and style position. On the other hand, there are results that reinforce the role of *S. l. cerasiforme* as the ancestor of tomato: A) Not all the *S. l. cerasiforme* accessions seemed to be admixtures according to the Structure analysis; this is the case for accessions collected in Ecuador and northern Peru. B) Most of the genome of the admixtures found in the Ecuadorian *S. l. cerasiforme* was similar to that of the non-admixtured *S. l. cerasiforme* from the Sucúa geographical group, so the Ecuadorian admixtures would always include the genome of an ancient non-admixtured *S. l. cerasiforme*. C) The Ecuadorian accessions, including those that are not admixtures, include plants that could easily be considered traditional small-fruited tomatoes. Moreover, the hybrid-*S. l. cerasiforme* hypothesis would imply that the ancestral transitional types of intermediate fruit size, created during the domestication process of *S. pimpinellifolium*, would have disappeared from the present variation observed in the Andean and Mesoamerican regions.

*S. l. cerasiforme* inhabited a large geographical region in pre-Columbian times, ranging from the Andes to Mesoamerica, and thus the domestication of tomato could have happened in any of these locations. No clear data exists in previous studies that definitively resolves the Andean and Mesoamerican domestication hypotheses [40]. One of the main arguments given by Jenkins [27] in favor of the Mesoamerican hypothesis was based on the great variability observed in the cultivated Mexican tomato and in the individuals with intermediate characteristics between the typical *S. l. cerasiforme* and *S. l. lycopersicum*. However, Jenkins also admitted that he did not have enough samples from the Andean regions to compare to those from Mesoamerica. This comparison was carried out by Rick and Fobes with isozymes [16]. They found that the Peru-Ecuador region showed the highest variability for the cultivated tomato. Moreover, Rick and Holle [17] also found high variation and a marked geographical structure in the Andean *S. l. cerasiforme*. Despite this evidence, they preferred the Mesoamerican hypothesis because they also found evidence of hybridizations between *S. pimpinellifolium* and *S. l. cerasiforme* in the Andean region that may have confounded the variability measurements.

Taking into account the aforementioned morphological and molecular data regarding Ecuadorian *S. l. cerasiforme*, we propose that a pre-domestication process took place in the Andean region that created small-fruited tomato varieties adapted to high rainfall climates. These varieties were very similar to the Ecuadorian *S. l. cerasiforme* accessions with bigger fruits that were included in this study and to those collected by Brücher in Venezuela, which he regarded as the ancestors of the cultivated tomato. However, given the complexity of the genetic variation detected in the Andean region, we cannot completely rule out the alternative possibility that the characters typical of the cultivated tomatoes found in Andean *S. l. cerasiforme* could have been introduced in more recent times from foreign materials.

This question could be better addressed if we had samples of ancient Mesoamerican and Andean tomato varieties, but this is not a trivial task. The Andean accessions collected as traditional tomato varieties in this study were later classified into two groups: those with smaller fruits were reclassified as *S. l. cerasiforme* and those with larger fruits seem to be derived from modern tomato breeds. The modern cultivars are eroding the traditional varieties, as was also noted by Rick [42], which probably included many cultigens that would be now classified as *S. l. cerasiforme*.

To settle this conjecture, it would be ideal to have archaeological evidence regarding tomato fruits in pre-Inca Andean cultures, but these remains have not been found in the Andean nor in the Mesoamerican regions [29], [30], [40], [44], [46]. Despite the lack of tomato-related archaeological remains, it is known that as of 4 to 5 millenniums ago, cultures with ceramics and agriculture were already living on the eastern slopes of the Ecuadorian Andes. However, only recently have archeologists begun the careful study of these cultures, such as that of Mayo-Chinchipe [47]. Despite these new excavations, it is important to remember that the high humidity of the region would impair the conservation of any remains of tomato cultivation.

Despite the controversy regarding exactly where the process started, all previous authors agree that the domestication of tomato was completed in Mesoamerica. The molecular and morphological data gathered in this study are also in accord with this hypothesis. In the PCA carried out without *S. pimpinellifolium*, the Mesoamerican *S. l. cerasiforme* is closest to the traditional Mesoamerican varieties. As has already been described, the polymorphism and heterozygosity of Mesoamerican *S. l. cerasiforme* is quite low, and thus the genetic diversity of the traditional varieties that descended from it is also quite low. One trait associated with the decrease in heterozygosity which occurred on the way from Andean *S. pimpinellifolium* to the worldwide spread of the cultivated tomato, passing through the Andean and Mesoamerican *S. l. cerasiforme*, is the style position, which has gradually become more inserted (Figure 5). Other characters that are of great importance are those related to fruit size: fruit length, width and weight and number of locules. *Solanum lycopersicum* var. *cerasiforme* also occupies an intermediate position with regard to these characters, and it is in the cultivated Mesoamerican tomatoes in which sizes comparable to those found in the cultivated tomatoes from around the world are found, with fruits that are markedly bigger than those found in pre-domesticated Andean tomatoes. Also worthy of mention is the presence of the semi-determinate and determinate growth in Mesoamerica. Only these accessions, along with one Peruvian accession, showed this trait. This character is controlled by the *sp* gene, and the first determinate plants were described in 1914 as a spontaneous mutation [48]. The determinate alleles present in Mesoamerica could have been native or, alternatively, they could have been introduced by modern determinate cultivars. The character was incorporated into commercial cultivars after the 1940s. In all probability, other characters also changed during the domestication process. Rick reviewed [16] this aspect in a study on the morphological diversity of Mesoamerican tomatoes, and Rodríguez et al. [49] analyzed genes related to fruit-shape diversity.

As has already been noted, when the Spaniards arrived in Mesoamerica, they found the cultivated tomato. The most accepted hypothesis states that they brought the tomatoes back to Spain from Mesoamerica [50]. The molecular data shown also agrees with this scenario: in the PCA analysis (Figure 2), the traditional European varieties are close to the Mesoamerican ones. This result is also compatible with the isozyme data presented by Rick and Fobes [16] and with the RAPD data gathered by Villand et al. [19]. The genetic differences found among the non-South and non-Mesoamerican tomatoes are low, which could be due to the short amount of time that passed between the popularization of tomato and the rise of significant trade between the different regions.

In conclusion, we hypothesize that, based on the molecular and morphological data presented, *S. l. cerasiforme* originated from *S. pimpinellifolium*. The tomato was later domesticated from *S. l. cerasiforme* in a process composed of several phases: first, a pre-domestication was carried out in the Andean region, during which *S. l. cerasiforme* developed a notable morphological diversity that included bigger fruits, which are even today being cultivated as small-fruited tomatoes. Those materials were then carried to Mesoamerica and it was there that the true domestication occurred, thus creating the traditional big-fruited tomato varieties. From there, the Spaniards took tomatoes to Spain and Italy, and from there they spread to the rest of the world.

These processes are, of course, far from being over; tomato germplasm is not static nowadays, as its adaptation to human needs did not end with its Mesoamerican domestication or with its worldwide conquest. In recent times, modern breeding has started a new phase in which almost all wild tomato relatives are being used for the genetic improvement of this crop. This is also clearly seen in the molecular data presented as well as in the results based on RAPDs [14], [19] and RFLPs [14] from previous studies. In the PCA, the modern cultivars appear quite different from the traditional ones, and the Structure analysis also detects new components in the genomic makeup of modern tomato. And finally, the genetic diversity of modern cultivars is markedly higher than that found in traditional varieties.

## Materials and Methods

### Plant material

The plant material used in this study comprised of 272 selected accessions. This sample included 63 accessions of *S. pimpinellifolium*, 106 of *S. l. cerasiforme*, 95 of *S. l. lycopersicum* and 8 derived from hybridization processes (Supporting Table S1). These accessions included 74 provided by different germplasm banks (AVRDC, CATIE, TGRC, USDA, VIR) as well as 194 collected by the Institute for the Conservation and Improvement of Agricultural Biodiversity (COMAV) and deposited in its own germplasm bank, and which represent a broad sample of the variation of *S. pimpinellifolium* and *S. lycopersicum*.

### Morphological characterization

The morphological characterization of 108 accessions (tagged in Supporting Table S1) was conducted in greenhouse in the spring-summer season of 2004, the data of which is available in Supporting Table S3. A completely randomized design was used with three plants per accession. Plants were grown in 12-liter pots with coconut fiber and were fertirrigated with the common dosages and regularity for tomato in our area. The remainder of the accessions were characterized in other assays, and even though their results were used to check the taxonomical classifications, they were not used in the Canonical Discriminant Analysis and do not appear in Figures 5 and 6.

Eleven quantitative traits and fifteen qualitative traits were recorded according to the descriptors for tomato developed by IPGRI [37] as well as several others selected from previous experience with wild-species management. The scored traits are listed in Table 2. The taxonomic classification of the accessions was assessed by means of their morphological characterization according to the following criteria (based on those proposed by Rick and Holle [17]):

*S. pimpinellifolium*: accessions collected in wild habitats, usually with ‘pimpinellifolium’-type leaves [37], round fruits of no more than 1.5 cm in diameter.
*S. l. cerasiforme*: In this work, we employed varied criteria, not only based on fruit size, to classify an accession as *S. l. cerasiforme*. We considered an accession to be *S. l. cerasiforme* if it had fruits of between 1.5 cm and approximately 5 cm in diameter. Although the fruits were mostly round and smooth, there were also ribbed and flattened ones [17]. In order to distinguish these last types from the small-fruited *S. l. lycopersicum* accessions, we also considered the type of location where they were found to grow and if they were weeds or cultivated plants. In any case, the modern, cultivated, commercial cherry tomato available worldwide has been considered and included as *S. l. cerasiforme*.
Traditional *S. l. lycopersicum* varieties. In this group, we included landraces and obsolete, non-improved, varieties. In general, they have morphologically less uniform fruits that may show scars and a large and fibrous core. They are less productive than the commercial varieties and show an irregular fruit set sequence.
Modern commercial *S. l. lycopersicum* varieties. Here we included varieties that have been genetically improved to be more uniform and productive.

**Table 2:** Quantitative and qualitative traits used in the morphological characterization of the accessions.

Plant	Flower and inflorescence	Fruit
**Quantitative traits**		
Plant height (cm)	Number of petals	Fruit weight (mg)
Number of leaflets	Sepal length (mm)	Fruit length (mm)
Number of small leaflets		Fruit width (mm)
		Number of locules
		Pedicel length abscission to fruit (mm)
		Pedicel length abscission to truss (mm)
**Qualitative traits**		
Plant growth type (dwarf, determinate, semi-determinate, indeterminate)	Inflorescence type (uniparous, multiparous)	External color of ripe fruit (yellow, orange, pink, red, other)
Leaf type (dwarf, potato, standard, pimpinellifolium)	Petal curvature (slight, intermediate, high)	Fruit cross-sectional shape (round, angular, irregular)
Leaflet border (entire, serrated, undulated)	Stile position (inserted, same level as stamen, slightly exserted, highly exserted)	Shape of pistil scar (dot, stellate, linear, irregular)
Stem pubescence density (sparse, intermediate, dense)		Skin color of ripe fruit (yellow, colorless)
Stem pubescence length (short, intermediate, long)		Width of pedicel scar (narrow, medium, wide)
Stem anthocyanin (dark, clear)		
Vein anthocyanin (dark, clear)		

After considering the morphological data, a reclassification of certain accessions was done. In some special cases, especially when distinguishing the traditional and commercial varieties, molecular characterization was used to complement the morphological one.

### DNA isolation and genotyping analysis

For each accession, genomic DNA was isolated from young leaves of one plant using the CTAB method [51]. DNA qualities were evaluated on agarose gels, and DNA concentrations were determined spectrophotometrically using an ND-1000 instrument (Thermo Fisher Scientific, Wilmington, North Carolina, USA).

The samples were genotyped using SolCAP's Illumina Bead Chips (Illumina, San Diego, California, USA) developed by the SolCAP project [31], [32]. Genotyping was performed using the TraitGenetics GmbH genotyping service (Gatersleben, Germany) according to the manufacturer's instructions for Illumina Infinium assaying (Illumina Inc., San Diego, CA, USA). Intensity data was processed using the Illumina GenomeStudio v.2011.1 software. The genotypes were called using a cluster file that was developed by SOLCAP and TraitGenetics. Further quality and reproducibility checks of SNP calls were done at TraitGenetics using duplicated DNA samples and standard lines.

### Data analysis

Prior to any molecular-based analysis, the set of markers to be considered was filtered, with markers with more than 10% missing genotypes or monomorphic (with 95% criteria) being removed. Additionally, when several markers were within 10 Kilobases of one another, all but one were removed.

The Principal Component Analyses (PCAs) conducted to study the pattern of genetic variation among the accessions were carried out with the filtered markers by using the smartPCA application included in the Eigensoft 3.0 package [33]. The AMOVA analyses were performed using the Arlequin software [34]. The AMOVAs were conducted taking into account the genotypes on a locus-by-locus basis and with 1000 permutations. A bayesian population classification based on the molecular data was done using the Structure software version 2.3.2.1 [35]. Structure runs were carried out with a burn-in of 20,000 and 100,000 repetitions with the number of populations (K) ranging from 2 to 19. The model used allowed admixture and took into account the physical location of each marker. Non-biased and observed heterozygosity as well as polymorphism were calculated using the *Genetix* version 4.05 software [52]. For the heterozygosity and polymorphism calculations, the mononorphic-markers filter was not applied.

The standard Köppen-Geiger climate classification displayed in the geographical maps was taken from Peel et al. [36].

The canonical discriminant analysis conducted on the morphological data was carried out using the *candisc* R library. Both the quantitative and the ordinal characters were used, whereas the nominal and binary ones were discarded for this analysis. The species classification was chosen as the classification variable.

The morphological distances were calculated using R and combining two distance matrixes [53], one for the quantitative and ordinal characters and the other for the binary and nominal characters. The Euclidean distance was used for the quantitative characters, and the Jaccard distance was computed on a table of dummy characters created from the transformation of the nominal and binary characters. The genetic Nei minimum distances were obtained using the *populations* software (http://www.bioinformatics.org/project/?group_id=84). A Mantel correlation test between the genetic and morphological distances was carried out with the *mantel.rtest* function of the *ade4* R package using 1000 permutations to assess the significance.

All charts were prepared by coding custom scripts, available upon request, that used the matplotlib Python library.

## Supporting Information

Figure S1 **Structure-Estimated Ln Prob of Data for different numbers of populations (K).**
(TIFF)

Figure S2 **Canonical discriminant analysis.** CDA analysis of the quantitative and ordinal morphological data. The projections of the accessions on the first two canonical variables are plotted. The colors used show the different genetic groups and match those in Figure 1. The markers differentiate the species: *S. pimpinellifolium* (triangle), *S. l. cerasiforme* (square) and *S. l. lycopersicum* (circle).
(TIFF)

Table S1 **Sample list and passport data.**
(XLS)

Table S2 **Genotypes determined with the SolCap array for all accessions.**
(CSV)

Table S3 **Morphological characterization data.**
(XLS)
