# Supplementary figures and images for: Variation Revealed by SNP Genotyping and Morphology Provides Insight into the Origin of the Tomato
Source: PLoS One. 2012 Oct 31;7(10):e48198. doi: 10.1371/journal.pone.0048198 (PMC3485194; doi:10.1371/journal.pone.0048198)

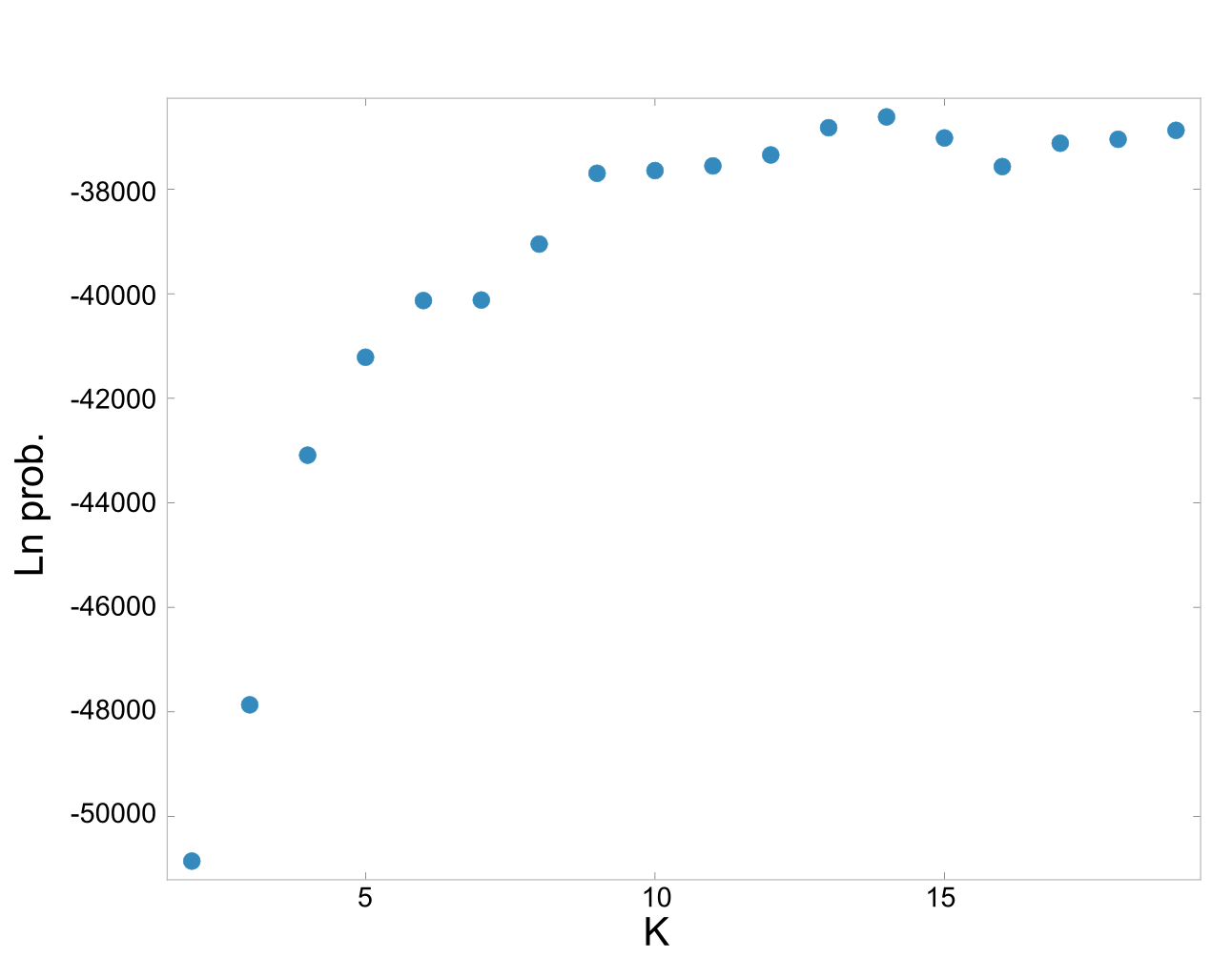

Supplement: Figure S1 — Structure-Estimated Ln Prob of Data for different numbers of populations (K). (TIFF) [file pone.0048198.s001.tiff]

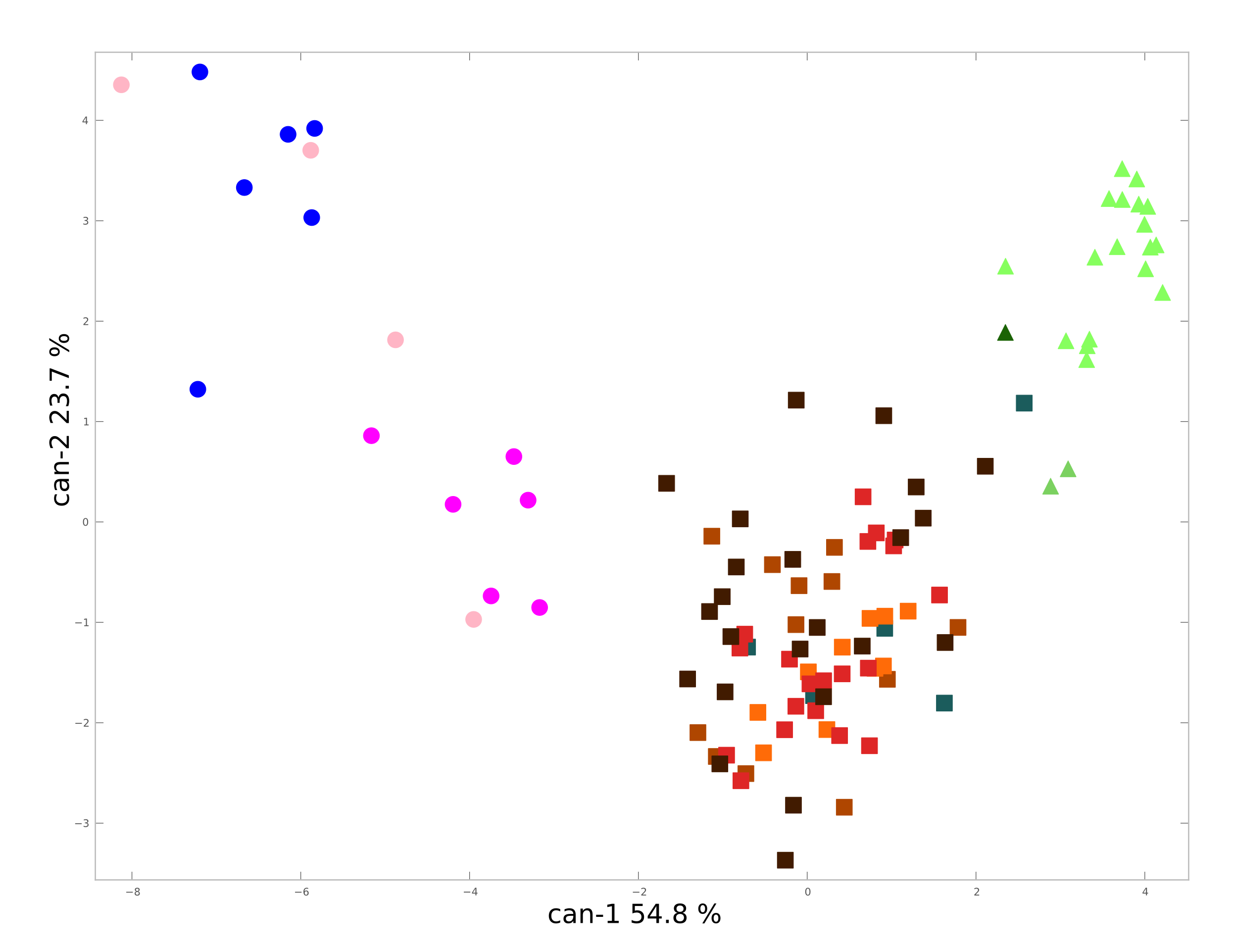

Supplement: Figure S2 — Canonical discriminant analysis. CDA analysis of the quantitative and ordinal morphological data. The projections of the accessions on the first two canonical variables are plotted. The colors used show the different genetic groups and match those in Figure 1. The markers differentiate the species: S. pimpinellifolium (triangle), S. l. cerasiforme (square) and S. l. lycopersicum (circle). (TIFF) [file pone.0048198.s002.tiff]
